# Phenotypic compatibility and specificity in genomic variant classification

**DOI:** 10.1038/s41431-024-01554-6

**Published:** 2024-02-13

**Authors:** Lina Basel-Salmon

**Affiliations:** 1 Raphael Recanati Genetic Institute, Rabin Medical Center – Beilinson Hospital, Petach Tikva, 4941492 Israel; 2 Faculty of Medicine, Tel Aviv University, Tel Aviv, 6997801 Israel; 3 Felsenstein Medical Research Center, Petach Tikva, 4920235 Israel

**Keywords:** Diagnosis, Diseases

## Introduction

Genome-wide sequencing for monogenic conditions for clinical purposes is used in two main settings: 1) preventive pre-conceptional, newborn, and healthy adult screening; and 2) diagnostic prenatal and postnatal testing. Reported genomic variants are categorized as diagnostic, secondary, incidental, or reproduction-related. Historically, diagnostic testing primarily targeted individuals identified by their phenotypes. However, recent years have witnessed a shift towards a “genotyping-first approach”, prompted largely by the decreasing costs of sequencing, particularly when balanced against the time clinicians spend characterizing phenotypes as well as the availability of clinicians to perform in-depth phenotyping.

However, as establishing phenotypic specificity and compatibility with a variant is essential for both classifying the variant and linking it to a patient’s clinical presentation, genotyping without prior phenotyping could have an impact on variant classification and case interpretation [1, 2]. Phenotype-genotype compatibility estimation might be further complicated by the suboptimal communication of detailed phenotypic information about the proband and the family members to the diagnostic laboratory [3]. Moreover, a clear definition of what constitutes a “specific phenotype” as well as a “compatible phenotype” is still lacking.

## Genomic testing in the diagnostic versus the preventive setting

Variant analysis is typically performed in the diagnostic setting when symptoms prompt genomic testing. The variants identified may be linked to a specific, clinically recognizable disorder or to a disorder with a nonspecific presentation. In the latter case, determining if a variant causes the observed phenotype (e.g., a de novo missense variant in a case of non-syndromic intellectual disability) is much more challenging. Data on prenatal phenotypes of many disorders are limited, and certain symptoms can be identified only postnatally. Symptoms in the proband that align with an identified variant may be previously unreported, suggesting phenotypic expansion, particularly in newly established gene-disease associations with few published cases. Symptoms seemingly unrelated to a candidate variant might be attributed to an additional monogenic disorder, chromosomal disorder, or multifactorial trait (e.g., benign familial macrocephaly unrelated to intellectual disability in the proband).

Although phenotypic information helps in using or adjusting the strength of variant classification criteria, it is less applicable in testing asymptomatic fetuses or individuals, when it is more difficult to determine variant pathogenicity, the likely inheritance pattern, and the specific disorder associated with a particular variant. In the preventive setting, individuals might display mild symptoms or have a relevant family history that goes unnoticed without thorough phenotyping before genetic testing. Additionally, often only the proband is tested, and cis/trans variant information and parent-of-origin data for imprinting-related disorders remain unknown.

## Phenotype-related criteria used in variant classification

Phenotype-related criteria are defined differently by different professional societies. Among the criteria proposed for classifying pathogenic variants in the American College of Medical Genetics and Genomics guidelines [4], the following are related to phenotype: PS2/PM6 – de novo occurrence with a compatible phenotype; PP4 – direct match to the phenotype. The PP4 criterion can be upgraded and coupled with segregation analysis, in scenarios with locus homogeneity [5]. Criteria taking the phenotype in individuals other than proband into account include: PS4 - affected individuals exhibit a compatible phenotype; PP1 - co-segregation is observed in individuals displaying a compatible phenotype; BS2 - presence in healthy individuals; BS4 - lack of segregation. In one study, PP4 was found to be the most inconsistently applied criterion in variant classification across nine molecular diagnostic laboratories [6]. ClinGen guidelines define four levels of phenotype specificity to be used when applying the PS2/PM6 criterion: 1) phenotype highly specific for gene; 2) phenotype consistent with gene but not highly specific; 3) phenotype consistent with gene but not highly specific and high genetic heterogeneity; 4) phenotype not consistent with gene [7]. In practice, categories 2 and 3 overlap, since nonspecific phenotypes are, as a rule, characterized by high genetic heterogeneity. Guidelines defined by the European Society of Human Genetics provide points for clinical variant grading depending on “if the gene does or does not fit the phenotype”. Penetrance is taken into account as well [8]. In the UK, as defined by the Association for Clinical Genomic Science, PP4 is used as a supporting criterion when the patient’s phenotype is consistent with a specific genetic etiology. It can only be used if all the known genes associated with the disorder have been analyzed using a highly sensitive method appropriate for the reported types of likely pathogenic/pathogenic variants, and if variants in the known genes explain the majority of cases with that clinical diagnosis. PP4 can be upgraded to moderate or strong after multidisciplinary team discussion: moderate, if additional, more specific phenotypic features are present, and strong, if there are pathognomonic findings (e.g., by enzymatic testing, muscle biopsy) [9].

## Clinical presentation and disorder specificity

The rarer an observed feature or combination of features, the more specific the phenotype. Very few symptoms can be considered pathognomonic, meaning that their presence indicates that a disorder is present beyond any doubt. The features or their combination might be rare, common, or very common. Below are examples of possible categories of disorders according to their phenotypic specificity:

A. Recognizable dysmorphic features, recognizable malformation patterns (e.g Cornelia de Lange syndrome 1 (OMIM # 122470))
B. Characteristic highly specific laboratory or radiological findings (absent dystrophin staining on muscle biopsy)
C. Characteristic compatible laboratory or radiological findings (elevated alkaline phosphatase in Hyperphosphatasia with impaired intellectual development syndrome 1 (OMIM # 239300), positive episignature with high or moderate confidence [10])
D. Involvement of multiple body systems, rare combination of features (retinitis pigmentosa and hearing loss in Usher syndrome type 1C (OMIM # 276904))
E. Involvement of multiple body systems, combination of common features (intellectual disability and epilepsy)
F. Involvement of one body system, rare feature (transverse limb defect)
G. Involvement of one body system, common feature (autism, short stature, cardiomyopathy)
H. Involvement of one body system, very common feature (cancer)

## Additional characteristics defining disorder specificity

In addition to the observed features, the following criteria are important in defining disorder specificity.

1. Prenatal vs postnatal presentation. Can the clinical presentation can be detected prenatally (e.g., Thanatophoric dysplasia, type I (OMIM # 187600)) or only postnatally (e.g., Rett syndrome (OMIM # 312750)). Prenatally, a disorder can manifest either early in pregnancy (e.g., Fraser syndrome 1 (OMIM # 219000) or in the third trimester (e.g., overgrowth-related syndromes). Postnatally, some or all features may be noticeable at birth (e.g., Kabuki syndrome 1 (OMIM # 147920) or evolve over time (e.g., dystonia or cardiomyopathy). Clinicians must determine whether the disorder is expected to be detectible clinically or by additional non-genetic evaluation at the time of testing based on the individual’s age. 
2. Severe vs mild phenotype. For some disorders, expressivity varies, with symptoms ranging from mild, with a minimal impact quality of life, to severe disability, even within the same family. For other disorders, disease severity is more consistent. Typically, recessive conditions show more uniform severity.
3. Progressive vs. non-progressive course. Whether symptoms are progressive or non-progressive is a key factor distinguishing disorders. For example, macrocephaly that is non-progressive usually indicates a benign familial condition or an association to *PTEN* gene (HGNC:9588) variants, whereas progressive macrocephaly might suggest a metabolic disease such as Canavan disease (# 271900). It is important to consider the progression of the disorder in the phenotypic description.

## Pedigree-related information and disorder specificity

1. Incomplete penetrance and variable expressivity. Knowing whether family members of the proband exhibit incomplete penetrance and variable expressivity aids in variant interpretation since these characteristics are more commonly associated with dominant inheritance conditions.
2. Gender-related differences in phenotypic presentation. Some disorders affect only males or only females. For example, abnormalities in fertility-related genes may impact the reproductive functions of only one gender. Pathogenic variants in the X-linked *PCDH19* gene (HGNC:14270) cause female-limited epilepsy. In X-linked dominant conditions, females might be affected more severely than males, or the disorder may be lethal in males.
3. Anticipation. The molecular basis for anticipation often involves repeat sequence expansions in the causative gene or genes associated with telomere length maintenance. Recognizing anticipation is crucial, as repeat expansions are more challenging to detect in exome sequencing or genome sequencing data. Furthermore, the presence of this phenomenon can support the likelihood that variants in a gene linked to telomere-associated diseases are causative.

## Conclusion

In summary, in order to improve variant classification and case interpretation, efforts should focus on creating categories of disorders by phenotypic specificity and assigning monogenic disorders to these categories. Additionally, it is necessary to adjust variant classification criteria for use in situations where phenotypic information is unavailable, either due to underreporting or due to the preventive nature of the testing.
